# Experimental Study of the Bending Properties and Deformation Analysis of Web-Reinforced Composite Sandwich Floor Slabs with Four Simply Supported Edges

**DOI:** 10.1371/journal.pone.0149103

**Published:** 2016-02-12

**Authors:** Yujun Qi, Hai Fang, Weiqing Liu

**Affiliations:** College of Civil Engineering, Nanjing Tech University, Nanjing 211816, China; Northwestern Polytechnical University, CHINA

## Abstract

Web-reinforced composite sandwich panels exhibit good mechanical properties in one-way bending, but few studies have investigated their flexural behavior and deformation calculation methods under conditions of four simply supported edges. This paper studies the bending performance of and deformation calculation methods for two-way web-reinforced composite sandwich panels with different web spacing and heights. Polyurethane foam, two-way orthogonal glass-fiber woven cloth and unsaturated resin were used as raw materials in this study. Vacuum infusion molding was used to prepare an ordinary composite sandwich panel and 5 web-reinforced composite sandwich panels with different spacing and web heights. The panels were subjected to two-way panel bending tests with simple support for all four edges. The mechanical properties of these sandwich panels during the elastic stage were determined by applying uniformly distributed loads. The non-linear mechanical characteristics and failure modes were obtained under centrally concentrated loading. Finally, simulations of the sandwich panels, which used the mechanical model established herein, were used to deduce the formulae for the deflection deformation for this type of sandwich panel. The experimental results show that webs can significantly improve the limit bearing capacity and flexural rigidity of sandwich panels, with smaller web spacing producing a stronger effect. When the web spacing is 75 mm, the limit bearing capacity is 4.63 times that of an ordinary sandwich panel. The deduced deflection calculation formulae provide values that agree well with the measurements (maximum error <15%). The results that are obtained herein can provide a foundation for the structural design of this type of panel.

## Introduction

Composite sandwich panels are composed of composite face sheets and lightweight core materials and offer beneficial features, such as light weight, high strength, corrosion resistance, good thermal insulation performance, impact resistance, and high designability [1–3]. They are applied in bridge structures, building structures and other engineering elements because of their economic, social and environmental benefits [4, 5]. A composite sandwich bridge deck exhibits a high bearing capacity and is easy to transport and assemble. The application of such designs in bridge reconstruction projects can reduce traffic disruption times by more than 80% [6] and significantly reduce construction costs. When used as floor slabs and roofs, composite sandwich panels can reduce the dead weight of a structure and mitigate the impact of earthquakes. They can effectively eliminate such issues as the high energy consumption and high pollution of the production processes of traditional building materials such as cement and steel [7].

Ordinary sandwich panels, under the action of an external load, can easily suffer interface failure and core material failure [8–10]; therefore, many scholars have proposed modified foam core materials to improve the mechanical properties of sandwich panels. The most common method is to adopt a foam with a higher density. This approach can effectively improve bending resistance and shear resistance while also increasing resistance to interfacial debonding [11]; however, it can also significantly increase the self-weight of a structure. Another type of reinforced core material is stitching foam, which is made by stitching together foam core material and face sheets using fiber; this approach can effectively prevent the occurrence of face sheet debonding [12]. In bending tests of a polyurethane (PU) foam sandwich panel, the flexural rigidity and maximum stress of the specimen were found to be 2.78 and 9 times those of ordinary sandwich panels, respectively [13]. However, the resin enrichment at pinhole cavities and fiber fracturing at the pinholes in stitching foam can affect its in-plane mechanical performance [14]. Novel Z-pinned sandwich panels are fabricated by laterally inserting already-cured composite pins at a certain angle into a sandwich panel, thereby connecting the face sheets and foam core material to effectively improve the panel’s mechanical performance [15, 16].

Unlike the foam core materials in ordinary sandwich panels, a web-reinforced composite sandwich panel contains a crisscross structure of composite stiffening ribs that lie parallel to the lateral loads sustained by the sandwich panel, which endows it with better mechanical performance. Manalo et al. studied two stress behavior types of sandwich panels under conditions of plane bending (in which the load direction is perpendicular to the plane of the composite panel) and lateral bending (in which the direction is parallel to the composite panel) [17]. The results revealed brittle shear failure of the core material in the former case, whereas the latter case resulted in side failure of the face sheet, whose destruction process exhibited obvious ductility. In addition, in-plane shear tests of the sandwich panels demonstrated that the composite panel parallel to the load direction can inhibit the generation and development of cracks in the core material, significantly improving both its bearing capacity and ductility [18]. Zi et al. studied the static behavior of an orthotropic bridge deck that was composed of glass-fiber-reinforced polymer (GFRP) and polyurethane foam and reported that the introduction of the foam improved the longitudinal response of the GFRP deck by 20% [19, 20]. Wang et al. conducted a four-point bending experiment to compare the performance of a sandwich panel with GFRP face sheets and a foam-web core with that of an ordinary sandwich panel, and the results indicated that the ordinary sandwich panel suffered interfacial debonding failure, whereas the web-reinforced composite sandwich panel did not suffer such failure, and that the bending capacity of the latter was five times that of the former [21]. Wu et al. conducted a quasi-static compression loading experiment on a foam-filled lattice composite panel, and the test results revealed that a maximum increase in peak strength of approximately 1600% compared to foam-core composite panels could be achieved by using lattice webs [22]. The abovementioned studies concerning web-reinforced composite sandwich panels have primarily focused on one-way bending and quasi-static compression performance as well as the related failure modes and bearing capacity. However, studies of two-way flexural performance and deformation computations are still lacking.

Therefore, this study was performed to investigate the bending mechanical properties of two-way web-reinforced panel and methods of computing their flexural deformation under lateral loading in the context of floor-slab applications. The experimental results may provide a basis for the engineering design of such panels in the future.

## Materials and Test Specimens

### Design and preparation of test specimens

For this experiment, five two-way web-reinforced composite sandwich panels and one ordinary polyurethane foam sandwich panel were designed and fabricated. The length and width of each specimen were both 1000 mm. The specimens were prepared with three different web heights, namely, 50 mm, 75 mm, and 100 mm, and three different web spacing values, namely, 75 mm, 125 mm and 175 mm. The parameters of each test specimen are summarized in Table 1.

**Table 1:** Parameters of the test components.

No.	Length of specimen	Width of specimen	Length of core	Height of core	Width of core	Number of layers in web	Fiber orientation in web	Number of layers in coat	Fiber orientation in coat
FS75	1000	1000	1000	75	1000	—	—	2	0/90°
SX50-75	1000	1000	75	50	75	2	-45/45°	2	0/90°
SX75-75	1000	1000	75	75	75	2	-45/45°	2	0/90°
SX100-75	1000	1000	75	100	75	2	-45/45°	2	0/90°
SX75-125	1000	1000	125	75	125	2	-45/45°	2	0/90°
SX75-175	1000	1000	175	75	175	2	-45/45°	2	0/90°

Note: “FS” indicates an ordinary polyurethane foam sandwich panel, and the numerical value following this letter code represents the height of the core material. “SX” indicates a two-way web-reinforced composite sandwich panel; the first numerical value following this letter code represents the web height, and the second represents the web spacing.

**Fig 1:**
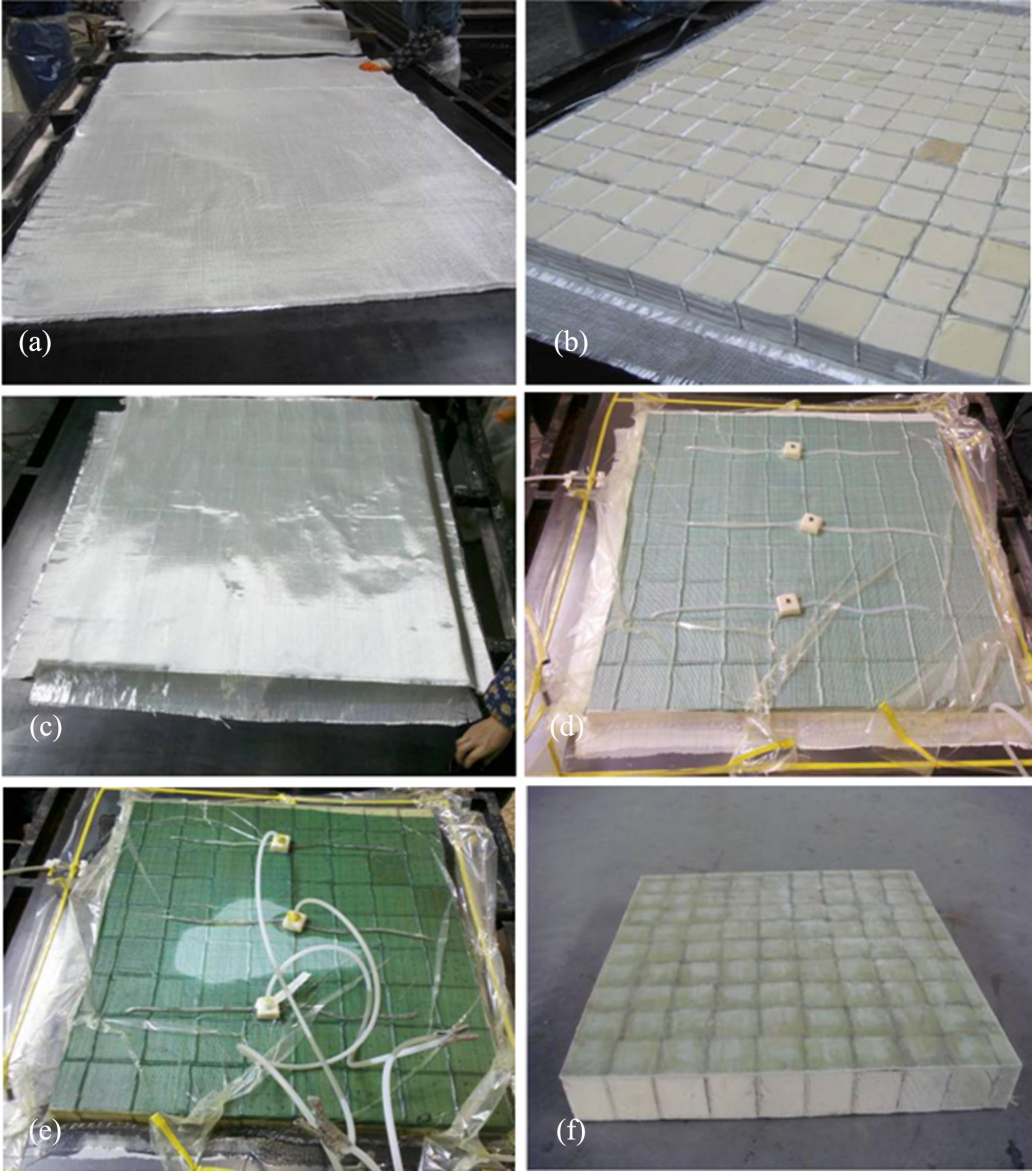
Process of sandwich panel preparation. (a) Laying of the lower layer of fiber cloth. (b) Laying of the core material. (c) Laying of the upper face sheet of fiber cloth. (d) Laying of the vacuum bag. (e) Resin infusion. (f) Cutting and shaping.

The raw materials that were used for the specimens included polyurethane foam, which has a density of 60 kg/m^3^; two-way orthogonal glass-fiber woven cloth, which has a surface density of 800 g/m^2^ and unsaturated polyurethane resin. The fiber orientation of the glass-fiber woven that was cloth used for the upper and lower face sheets was 0/90°, and the fiber orientation of the glass-fiber woven cloth in the web was -45/45°. All the components were prepared by using the vacuum infusion process, which consists of the laying of the fiber cloth, the cutting and laying of the core material, the laying of the vacuum bag, resin infusion, molding and demolding (Fig 1). The primary advantage of the process is the ability to achieve the one-step molding of composite face sheets, webs and core materials with good structural integrity [21, 22].

Compared to an ordinary sandwich panel, when the web-reinforced composite sandwich panel in this study was made by the above vacuum infusion process, the glass-fiber woven cloth between the foam pieces could channel the resin and could easily pass from one end of the panel to the other through the fiber cloth between the foam blocks. In addition, the core of the web-reinforced composite sandwich panel herein included many rather small foam blocks, different from the large chunks of foam core in ordinary sandwich panels. During vacuum infusion, these small foams might move and thus influence the overall dimensions of the sandwich panel. Therefore, pre-vacuum pumping was conducted first, without the infusion of resin, during the actual vacuum infusion. While vacuum-pumping, small foam blocks were adjusted to the correct positions. When the internal air pressure of the vacuum bag was smaller than 0.1 atm, all the foam blocks and fiber cloths, which could keep their predetermined shape, were compressed together. At this time, the resin could be infused. The resin could be demolded after curing.

### Material properties

#### Glass-fiber-reinforced polymer (GFRP) panels

**Fig 2:**
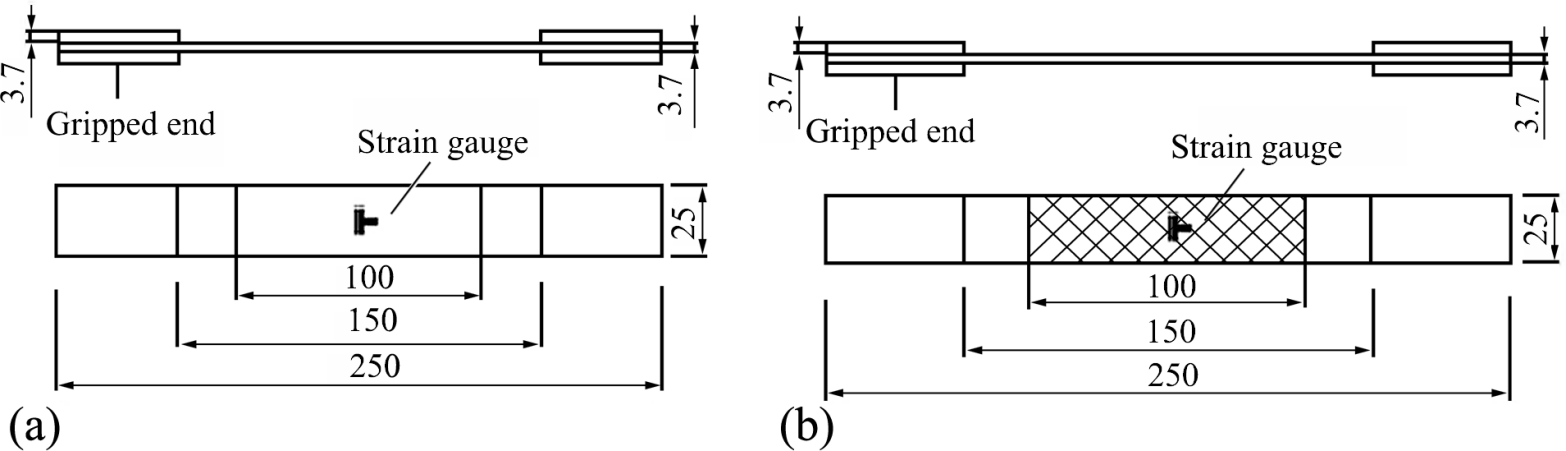
Specimens for the GFRP tests. (a) Specimen size for the tensile tests of the face sheets and webs. (b) Specimen size for the longitudinal and transverse shearing tests of the webs.

The GFRP specimens for the tensile tests were prepared (Fig 2) in accordance with the Test Method for the Tensile Properties of Glass Fiber-Reinforced Plastics (GB/T 1447–2005). A universal testing machine was used for the loading tests. The test results are shown in Table 2.

**Table 2:** Results of face sheet tensile tests.

No.	*h* (mm)	*b* (mm)	Tensile strength *f*_*t*_ (MPa)			Elasticity modulus *E*_*f*_ (GPa)		
			Test value	Average value	Variable coefficient (%)	Test value	Average value	Variable coefficient (%)
1	3.16	24.9	305.2			19.99		
2	3.06	24.08	290.9			20.28		
3	3.10	26.04	337.7	322.9	7.6	20.45	20.95	6.4
4	3.18	25.22	351.3			20.74		
5	3.26	25.02	329.4			23.30		
1	2.02	24.04	290.4			6.44		
2	2.06	24.20	289.8			5.39		
3	2.28	24.26	290.3	296.3	3.1	6.18	6.41	10.6
4	2.08	24.08	300.5			7.02		
5	2.14	24.04	310.5			7.03		

#### Foam core material

**Fig 3:**
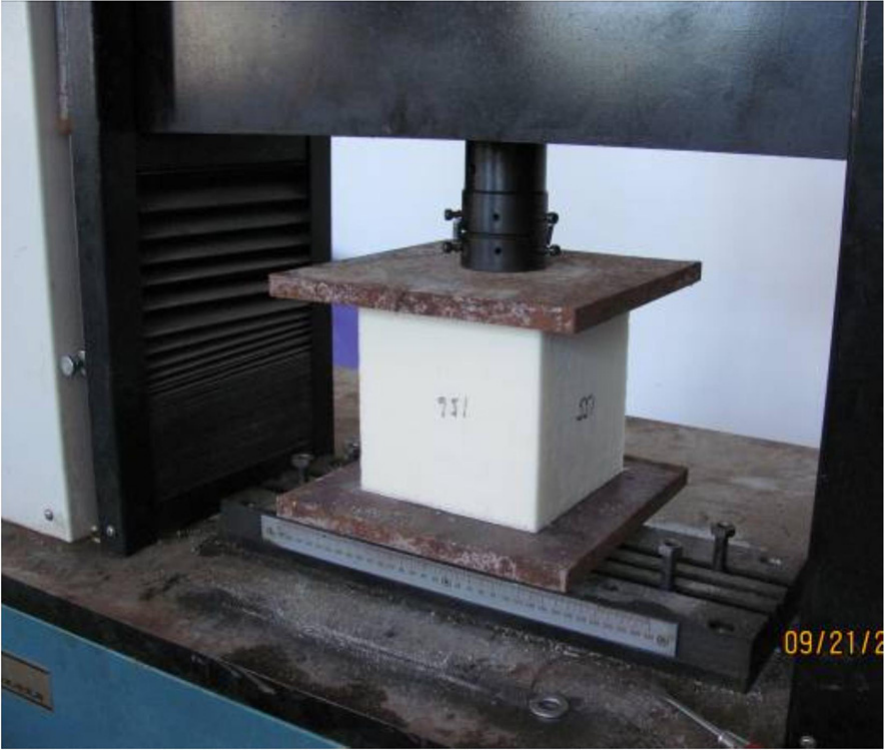
Foam compression test.

**Fig 4:**
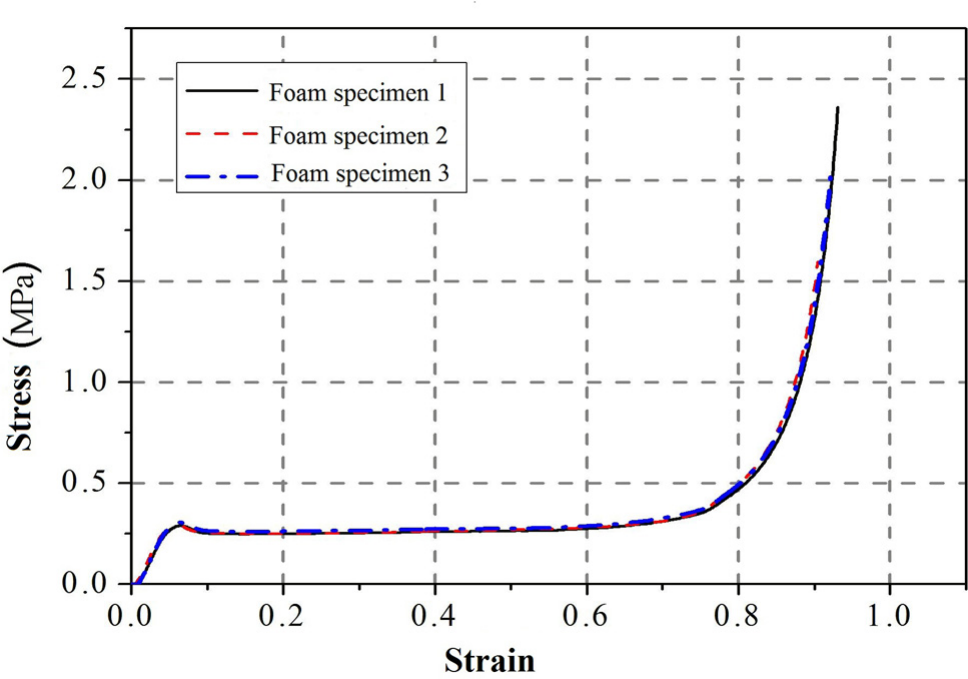
Stress-strain curves from flat crush tests of the foam core material.

Following the Test Method for the Mechanical Performance of Rigid Polyurethane Foam Plastics (GJB1585A-2004), flat crush tests of the foam core material were conducted to determine the compressive elasticity modulus and Poisson's ratio. Each foam specimen was a 150 mm×150 mm×150 mm cube, and an electrical universal testing machine was used for the compression tests. A 2-cm-thick steel plate was placed on each loading end. The testing device is depicted in Fig 3. The stress-strain curves of the foam core material are shown in Fig 4, where the elasticity modulus *E* is 5.534 MPa and Poisson's ratio *v* is 0.3.

### Test setup and measurements

**Fig 5:**
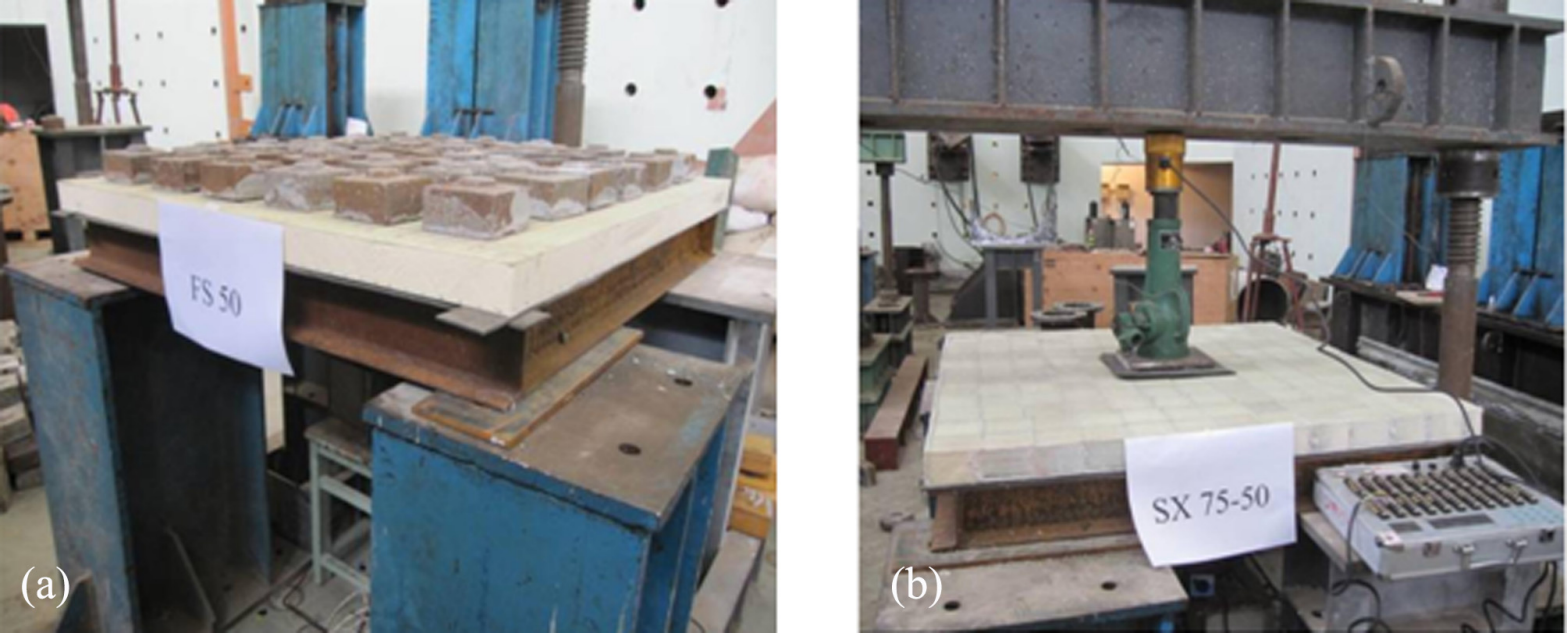
Test loading device. (a) Uniform loading. (b) Concentrated loading.

Each sandwich panel test specimen was placed directly on a 1 m×1 m square base that was welded from four steel channels with a width of 45 mm and a height of 100 mm (Fig 5). The four corners of the square base were fixed to the top ends of the four square steel buttresses. A 3-mm-thick rubber gasket was placed between the specimen and the steel channels to prevent local damage to the specimen at its base.

Five displacement meters were placed evenly along the horizontal symmetry axis of the specimen’s lower face sheet to measure the vertical displacement of the specimen, and a 5AA strain gauge was placed at the center of underside of the panel to measure the strain on the GFRP.

### Loading plan

The loading of each specimen was performed in two phases: a uniformly distributed loading phase and a midpoint-localized loading phase [23, 24].

First, weights were placed on the board to apply a uniformly distributed load. During the loading process, the total mass of the applied weights was recorded at each step, and the readings from the strain gauge and the displacement meters placed on the underside of the sandwich panel were collected after each load was applied. Each set of 36 3.5-kg weights corresponded to the next loading level, and there were 8 levels in all. The equivalent uniformly distributed load at each level was 1.26 kN/m^2^, and the maximum uniformly distributed load on the panel after all 8 levels of load were applied was 10.08 kN/m^2^. After each level of load was applied, the specimen was left undisturbed for five minutes for static observation. Once the sandwich panel achieved stability after deformation, the next level of load was applied. To prevent the flexure of the board from causing the adjacent weights to come into contact with each other, the weights were loaded at a spacing of no less than 5 cm.

After the uniformly distributed loading test described above, a concentrated load was applied to the specimen. During the loading process, readings were collected from a force transducer as well as the strain gauge and the displacement meters placed on the underside of the sandwich panel. The loading position was a 100 mm×100 mm square area near the center of the panel. A 100 mm×100 mm×20 mm thick steel plate was placed on this square area as a loading plate, upon which a jack was directly applied. To prevent local failure from excessive strain concentration near the loading area, hard rubber cushions with dimensions of 150 mm×150 mm×6 mm were placed between the loading area on the panel and the loading plate.

## Test Results and Discussion

### Uniform loading

Islam and Aravinthan conducted uniformly distributed loading tests under conditions of either two or four simply supported edges by using a type of ordinary sandwich panel with modified phenolic foam as the core material and GFRP as the face sheets [23]. The experimental results yielded load-deflection curves that were approximately linear under both types of boundary conditions.

The experimental results obtained in that study were essentially consistent with the results reported here; namely, during the process of uniformly distributed loading, the deflection of the panel’s underside increased with increasing load. No other obvious phenomena were observed. The appearance of the specimen exhibited no obvious change. The load-central point deflection curve of the specimen exhibited an essentially linear increase during the loading process, indicating that throughout the entire load process, all specimens were in a state of linear elastic stress and there was little or no internal damage.

**Fig 6:**
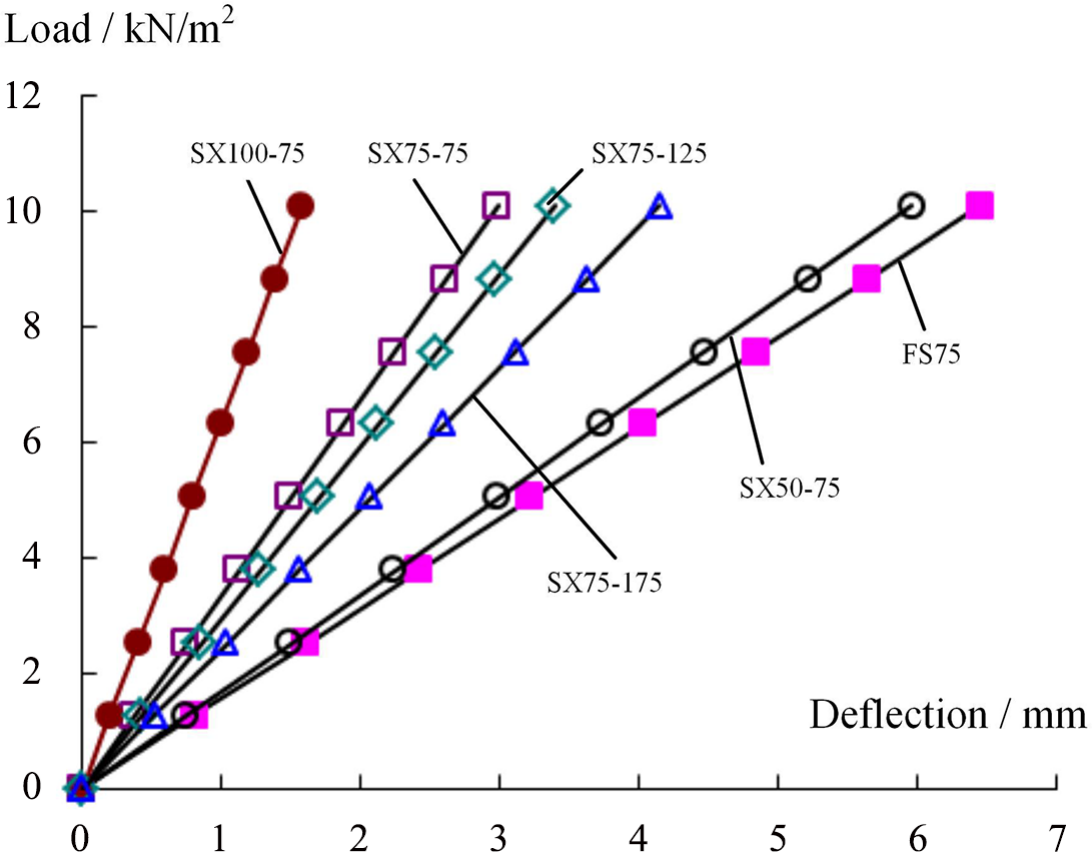
Load-central point vertical displacement curve.

Fig 6 shows that the web parameters exert a notable impact on the specimen’s midspan deflection:

1. A smaller spacing between webs resulted in a smaller deflection. The largest observed midspan deflection of 6.45 mm was recorded for the specimen without webs (FS75). The maximum midspan deflections of the web-reinforced specimens SX75-75, SX75-125, and SX75-175 were 2.98 mm, 3.39 mm and 4.14 mm, respectively, corresponding to 64.2%, 52.6% and 46.2%, respectively, of the value for the webless specimen.
2. Higher webs also resulted in smaller deflections. Among the specimen SX50-75, SX75-75, and SX100-75, whose web spacing values were all 75 mm, the maximum deflections were 5.96 mm, 2.98 mm and 1.58 mm, respectively. The deflections of the specimens with web heights of 75 mm and 100 mm were 50% and 26.5%, respectively, of that of the specimen with a web thickness of 50 mm.

These results indicate that placing webs in a webless specimen or reducing the spacing between the webs and increasing the web height in a web-reinforced specimen can effectively increase the flexural rigidity and reduce the vertical deformation of the component.

### Concentrated loading

#### Experimental phenomena and failure modes

The five specimens exhibited two typical failure modes during the concentrated loading process: debonding failure of the upper face sheet and fracture failure of the upper face sheet (Table 3).

**Table 3:** Experimental results for specimens with different web heights.

Specimen	Load (kN) at which cracking sounds were heard	Failure load (kN)	Maximum midspan deflection (mm)	Debonding degree of upper face sheet
FS75	8.0	18.3	16.5	Serious
SX75-75	54.2	83.3	27.9	None
SX75-125	34.4	65.0	29.1	Rather slight
SX75-175	22.0	49.8	31.2	Serious
SX50-75	30.0	53.2	40.5	None
SX100-75	62.5	108.8	25.7	Rather slight

(1) Upper face sheet debonding failure

The specimens that exhibited this mode of failure were the webless specimen, FS75, and specimen SX75-175, which had a rather large spacing between webs.

In the case of the webless specimen, during the initial loading stage, no apparent change was observed. Once 8.0 kN of load had been applied, slight cracking sounds were heard from the fiber. At this time, the loading process was halted for observation. It was found that the load and deflection both remained the same. Upon increasing the load to 18.3 kN, a loud snapping sound was suddenly heard, and the load abruptly decreased by a large amount. At this time, the specimen was observed once again. A large raised area was apparent, although no cracks were visible to the human eye. Knocking on the specimen produced a sound like that of an empty drum, indicating that debonding between the upper face sheet and the core material had occurred over a large area. It was impossible to continue loading the specimen. There was no obvious indication of failure prior to its occurrence; therefore, this was a brittle failure mode.

**Fig 7:**
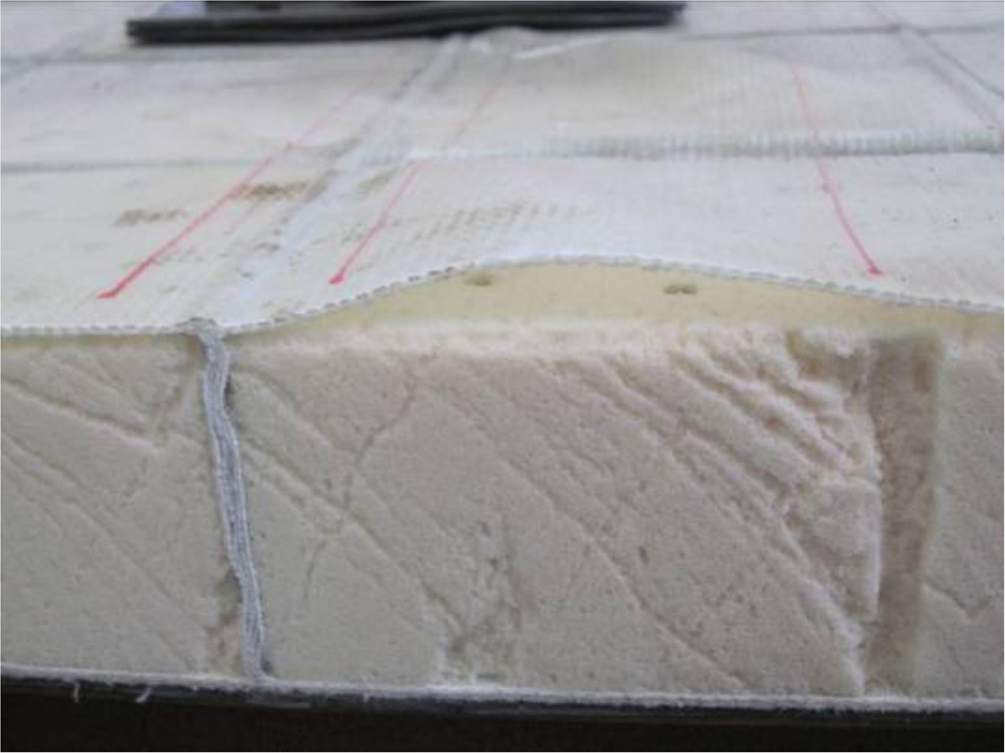
Photograph of the failure of specimen SX75-175.

The behavior of specimen SX75-175, with a rather large spacing between its webs, was similar to that of the webless specimen during the initial loading stage. When the load had increased to a large extent (38.2 kN), slight debonding of the upper face sheet occurred, and simultaneously, a few white cracks appeared near the loading surface. As the loading process continued, the surface detachment zone spread to surrounding regions, finally leading to a large area of debonding failure (Fig 7).

(2) Fracture failure of top panel

**Fig 8:**
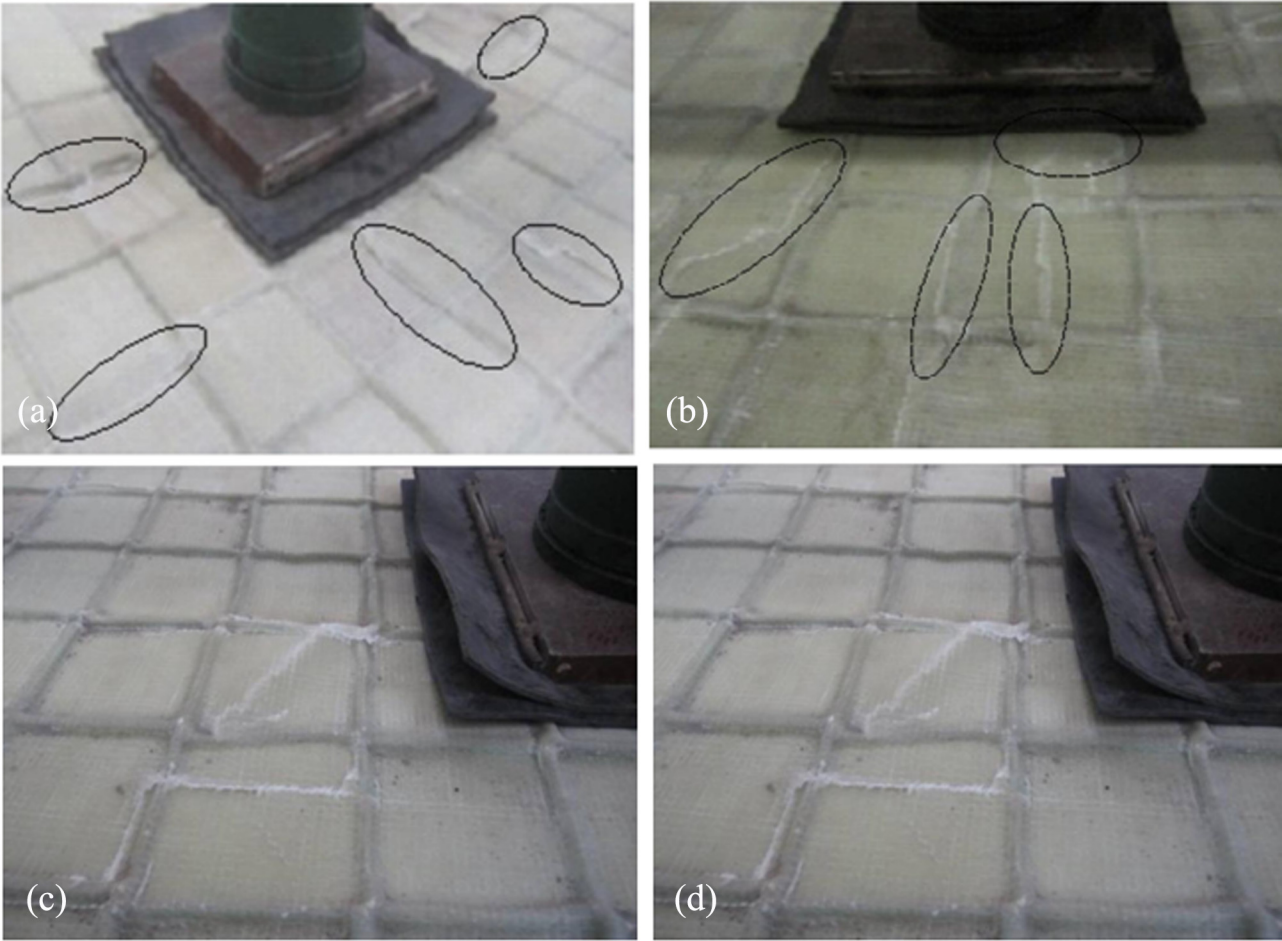
Fracture failure of the upper face sheet. (a) SX50-75. (b) SX75-75. (c) SX100-75. (d) SX75-125.

This failure mode was observed in specimens SX50-75, SX75-75, SX100-75 and SX75-125, all of which contained webs (Fig 8).

During the initial loading stage, the behaviors of these specimens were similar to those described above. However, the load at which slight cracking sounds were first heard was larger than in the case of either of the above-mentioned components. As the loading continued, a rather large area of local fiber fracture and cracking sounds from the resin appeared successively. Meanwhile, several white cracks parallel to the webs emerged on the upper face sheet, indicating obvious resin cracking in the upper face sheet of the GFRP plate. As the loading process continued, new cracks appeared, and the previous cracks continued to expand. When the ultimate load was reached, the specimen suddenly emitted an explosive “booming” sound, and several fractures appeared on the upper face sheet of the GFRP plate. In several locations, slanting wrinkles and even ladder-like fractures appeared. The specimen could sustain no further loading, indicating that the specimen had suffered its final failure.

Wang et al. conducted four-point bending experiments on ordinary sandwich panels and composite sandwich panels that were reinforced with two-way webs, and the results showed that interfacial debonding failure occurred between the core material and the face sheet for the ordinary sandwich panels [21]. Upper face sheet compression failure occurred in a majority of the composite sandwich panels reinforced with two-way webs; however, when the web thickness was rather small, shear failure of the core material occurred. The failure modes of the specimens in the present experiments were essentially identical to the research results of Wang et al. However, because the sandwich panel was simply supported at all four edges, it was impossible to observe shear failure of the core material.

The results of the aforementioned experiment demonstrate that sandwich panels that are not reinforced by webs are apt to undergo flexion deformation toward the outer surface under transverse load, which ultimately leads to face sheet debonding failure because of the weak constraints applied by the core material to the GFRP plate. For a web-reinforced sandwich panel, the webs apply an effective out-of-plane constraint on the upper face sheet of the GFRP plate. This constraint limits the development of out-of-plane deformation and inhibits the debonding failure of the upper face sheet of the GFRP plate, thereby increasing the ultimate loading capacity of the sandwich panel. However, when the spacing between webs is sufficiently large, the constraining role of the webs is compromised, which may lead to local debonding failure. Therefore, the spacing between webs has a significant impact on the failure modes of sandwich panels.

#### Load-displacement curves

**Fig 9:**
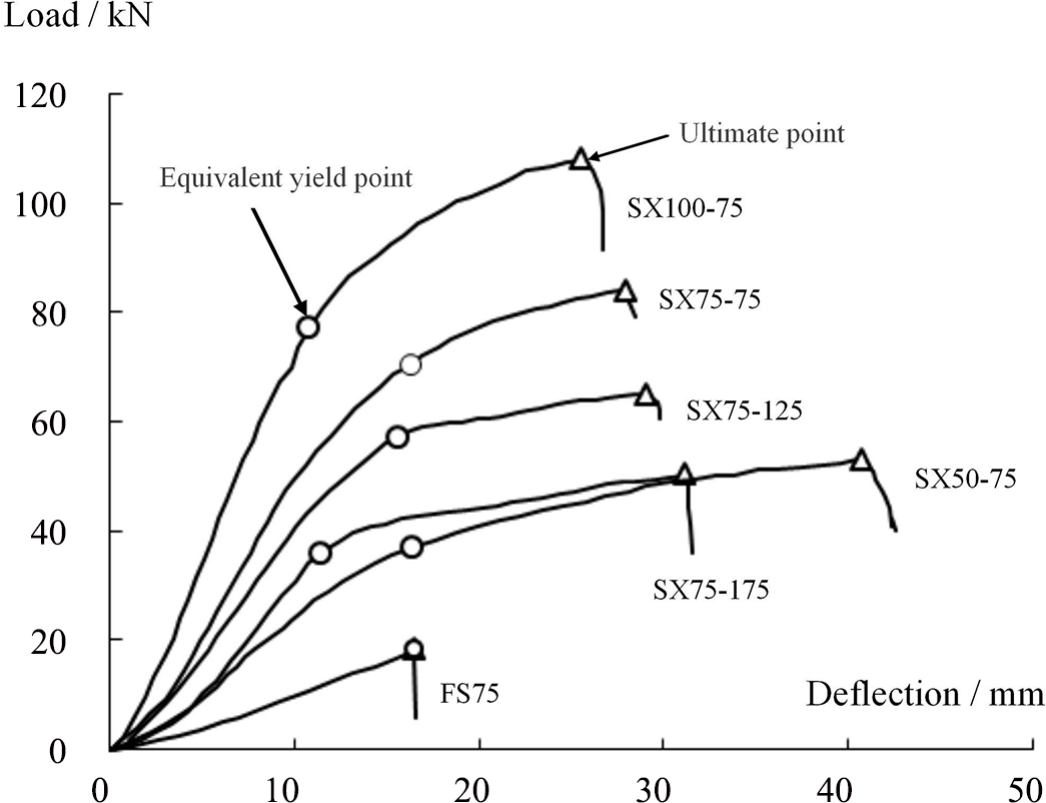
Load-deflection curves of the specimens.

The load-midspan displacement curves of the specimens are shown in Fig 9. Because of the greater space between loading devices during the initial loading stage, the slope of the load-deflection curve in this stage was quite small (load<10 kN or deformation<4 mm). With further loading, the spacing between weights decreased and the slope of the load-deflection curve increased. Once the deflection reached 4–5 mm, the slope gradually stabilized. During further loading, the webless sandwich panel and the web-reinforced sandwich panels exhibited the following differences in behavior:

1. For the web-reinforced composite sandwich panels, the midspan deflection and load exhibited nearly linear growth. When the ultimate load was reached, the load suddenly decreased; the sandwich panel underwent face sheet debonding failure, which was a brittle failure, and the sandwich panel abruptly lost its loading capacity.
2. For the web-reinforced composite sandwich panels, the load-displacement curve exhibited three obvious loading phases. The first phase was the linear loading phase, during which the panels underwent no obvious change. Once the load reached a certain value, the curve showed an obvious transition, after which the slope began to decline, as it entered the second phase. At this time, fiber cracking sounds began to occur, and simultaneously, white cracks appeared and gradually spread on the upper face sheet of the GFRP plate. When the ultimate load was reached, the GFRP plate on top of each sandwich panel underwent apparent fracture, and the loading capacity instantaneously decreased considerably, leading to the final failure of the sandwich panel.

Dawood conducted two-way bending experiments that involved the central single-point loading of 3-D GFRP sandwich panels with through-thickness fiber insertions to obtain the corresponding load-midspan deflection curve [25]. The specimen thicknesses in this experiment were quite small (the materials were available only in thicknesses of 25 mm and 50 mm); therefore, at large deflection, the face sheet exerted a sort of film effect, causing the slope of the curve to increase beyond the initial slope. However, the entire curve could be approximated by a linear change when the deflection was sufficiently large. When the component finally failed, the bearing capacity abruptly decreased. Islam and Aravinthan considered a type of ordinary sandwich panel with modified phenolic foam as the core material and GFRP face sheets under conditions of either two or four simply supported edges and performed concentrated loading experiments, which also indicated that the load-deflection curves of the specimens exhibited an approximately linear change followed by sudden brittle failure [23]. The experimental results discussed above are consistent with the load-midspan deflection curve recorded in this study for specimen FS75, an ordinary sandwich panel without web reinforcement.

In single-bend tests, Manalo et al. obtained the load-displacement curves for a sandwich panel under conditions of lateral bending (with the load perpendicular to the face sheet) and in-plane bending (with the load parallel to the face sheet; in this case, the face sheet played an equivalent role to that of the webs) [26]. The results indicated that the former exhibited typical elastic-brittle failure, identical to that of the webless specimen FS75 in this experiment. The latter demonstrated a rather high bearing capacity, and after failure, the specimen exhibited some ductility, consistent with the results for the web-reinforced specimens in this study.

The above experimental results indicate that the load-displacement curve of a webless two-way sandwich panel can be approximated as a linear relationship and that its ultimate failure manifests as sudden brittle failure. The results are similar to those for a single-bending sandwich panel. By contrast, two-way web-reinforced composite sandwich panels exhibit a higher bearing capacity and better ductility after failure. The primary reason for this difference lies in the fact that the webs reinforce the interfacial coupling between the core material and the face sheet and inhibit the expansion of cracks in the core material [21].

The following sections present analyses of the beneficial influence of webs on the mechanical properties of a sandwich panel in terms of bearing capacity and ductility.

#### Influence of webs on the bearing capacity of a sandwich panel

Let the equivalent yield load *P*_y_ be defined as the load that corresponds to the point on a component’s load-displacement curve with the largest change in slope, and let the limit load *P*_u_ be defined as the largest load on the component’s load-displacement curve (Fig 9). The experimental results show that the web spacing and web height exert a significant influence on the bearing capacity of the specimen.

**Fig 10:**
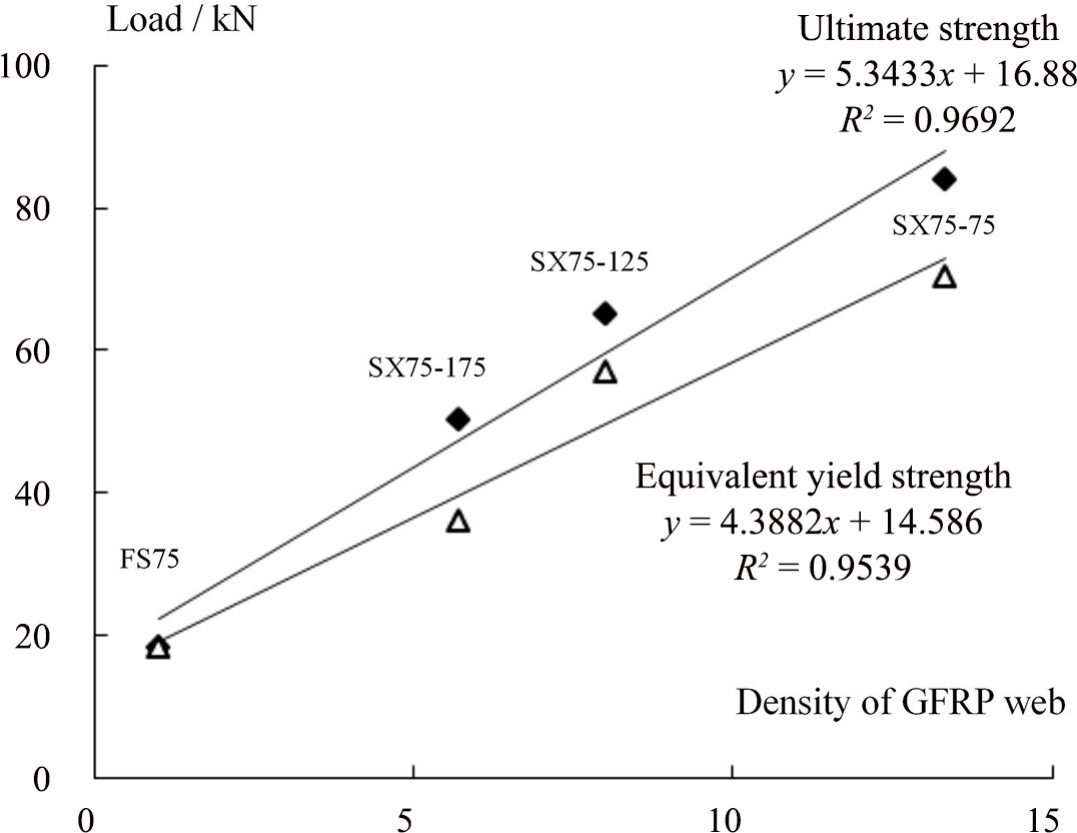
Relationship between the specimen load and web density.

**Fig 11:**
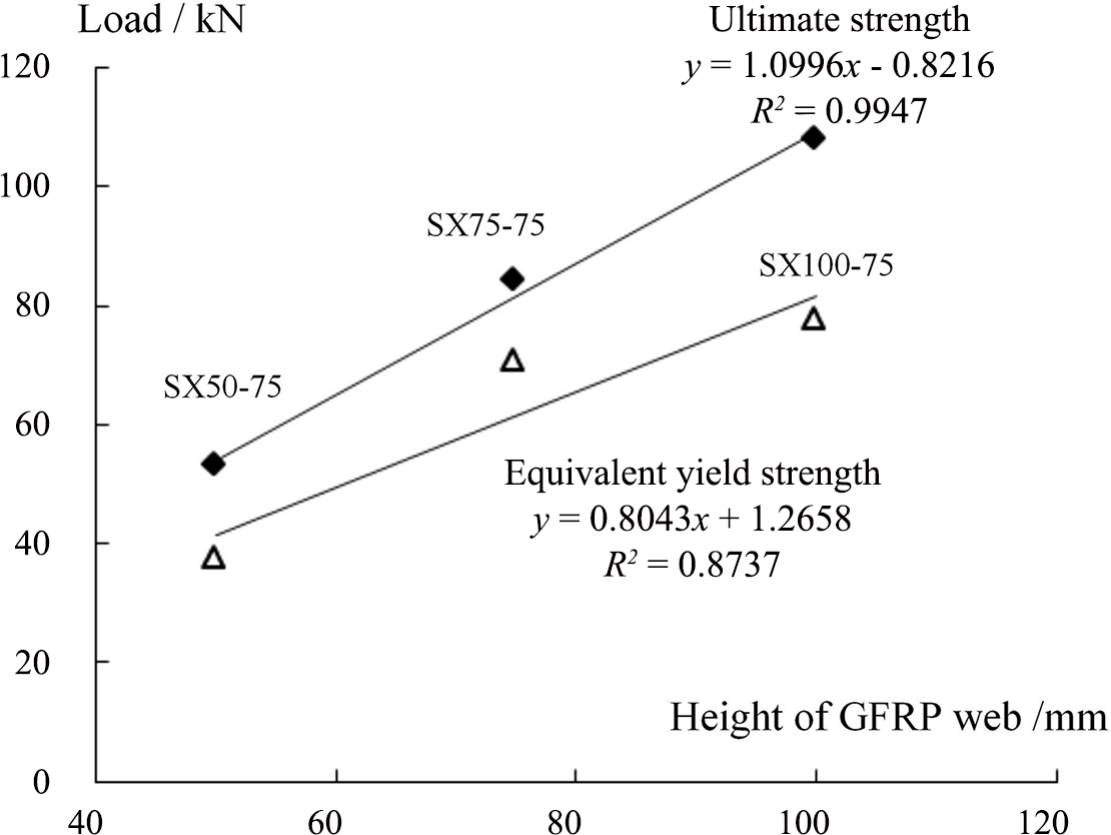
Relationship between the specimen load and web height.

Fig 9 shows that the equivalent yield loads for specimens SX75-175, SX75-125 and SX75-75, whose web heights were all 75 mm, were 35.98 kN, 56.90 kN and 70.41 kN, respectively, corresponding to 1.98, 3.14 and 3.88 times the yield load of specimen FS75 (18.13 kN). The limit loads were 50.23 kN, 65.06 kN, and 83.97 kN, respectively, corresponding to 2.77, 3.59 and 4.63 times that of the webless component (18.13 kN). These results indicate that the introduction of a two-way web into a sandwich panel with four simply supported edges could significantly increase its bearing capacity for a load that is perpendicular to the board. Furthermore, smaller web spacing produced a stronger effect. In the literature [21], single-bend tests of web-reinforced composite sandwich panels with web heights of 100 mm, 75 mm and 50 mm have been reported. The results revealed that the limit load of the former was increased by 36.7% and 49.8%, respectively, compared with those of the latter two, consistent with the results of this study. Meanwhile, for specimens SX50-70, SX75-75, and SX100-75, whose web spacing values were all 75 mm, the equivalent yield load and limit load increased with increasing web height. For the specimen with a web height of 100 mm, its equivalent yield load and limit load were 77.28 kN and 107.98 kN, respectively, which were 2.08 and 2.04 times those of the specimen with a web height of 50 mm. These results were different from those of Wang et al., who conducted four-point bending tests of two types of sandwich panels with web spacing values of 75 mm and 100 mm [21]. This difference can be explained by noting that the sandwich panels investigated in this experiment were subjected to two-way bending, meaning that the internal force distribution was more complicated than that in a single-bending sandwich panel and that the role of the two-way web structure was greater. Let the web density *a* be defined as the number of webs contained in a 1 m panel width; then, *a* = 1000/D, where D is the web spacing and the unit is mm. Here, *a* = 1 represents the case without webs. Figs 10 and 11 show the variations in the equivalent yield load *P*_y_ and the ultimate load *P*_u_ with the web density *a* and the web height, respectively. The results reveal that *P*_y_ and *P*_u_ both notably increase with increasing web density and height. The results of linear regression show that this variation can be regarded as a linear change.

#### Influence of webs on the ductility of a sandwich panel

On the load-displacement curve, the displacement that corresponds to the equivalent yield load *P*_y_ is the equivalent elastic displacement *f*_y_, and the displacement that corresponds to the limit load *P*_u_ is the limit displacement *f*_u_; then, the equivalent ductility coefficient *β* of the sandwich panel can be defined as *f*_u_ / *f*_y_. Larger values of this quantity indicate greater ductility of the component. The equivalent ductility coefficients of the 75-mm-thick specimens are shown in Table 4. The equivalent ductility coefficient of the sandwich panel without web reinforcement is 1, indicating that when brittle failure occurs, the specimen exhibits no ductility. For the web-reinforced sandwich panels, the equivalent ductility coefficient increases with decreasing web spacing.

**Table 4:** Test result comparison.

Specimen	Equivalent yield load *P*_y_ (kN)	Equivalent elastic displacement *f*_y_ (mm)	Limit displacement *f*_u_ (mm)	Equivalent ductility coefficient β = *f*_u_/*f*_y_	Maximum tensile strain of the lower panel under the equivalent yield load (MPa)
FS75	18.13	16.50	16.50	1.00	22.8
SX75-175	35.98	11.44	31.22	2.73	40.2
SX75-125	56.90	15.58	29.07	1.87	56.1
SX75-75	70.41	16.28	27.94	1.72	157.9
SX50-75	37.07	16.43	40.49	2.46	134.8
SX100-75	77.28	10.83	25.59	2.36	71.7

The discussions presented above demonstrate that composite sandwich panels with web reinforcement outperform ordinary sandwich panels without web reinforcement in terms of limit bearing capacity and ductility. The web spacing has a significant impact on the limit bearing capacity and ductility of sandwich panels. A reduction in web spacing can improve the limit bearing capacity of a sandwich panel. However, it can also simultaneously decrease the ductility of the sandwich panel. Therefore, the proper web spacing should be selected according to the actual bearing capacity and ductility requirements of the sandwich panel application.

## Deformation Analysis

Generally, the elastic modulus of GFRP is rather small, being only 1/10~1/7 that of steel or even less, and the material also suffers rather large shear deformation under lateral bending [17], causing composite sandwich floor slabs with GFRP as a face sheet to exhibit larger flexural deformation, which is undesirable during the normal use of a structure. Therefore, many scholars have studied the flexural deformation of this material and methods of computing it [17, 21, 27]. However, most of these studies have been confined to one-way slabs, and there is a lack of computational research on the flexural deformation of two-way slabs. Dawood studied the deformation of sandwich panels without web reinforcement under two-way bending [25]; however, this research was limited to concentrated loading, and uniformly distributed loads were not considered. Therefore, in this study, the continuous assumption was adopted to develop a model of a two-way web-reinforced composite sandwich panel, which was then used in simulations to derive the solution for the flexural deformation of such panels as a reference for their application in floor slab engineering.

### The continuous assumption and the mechanical model

**Fig 12:**
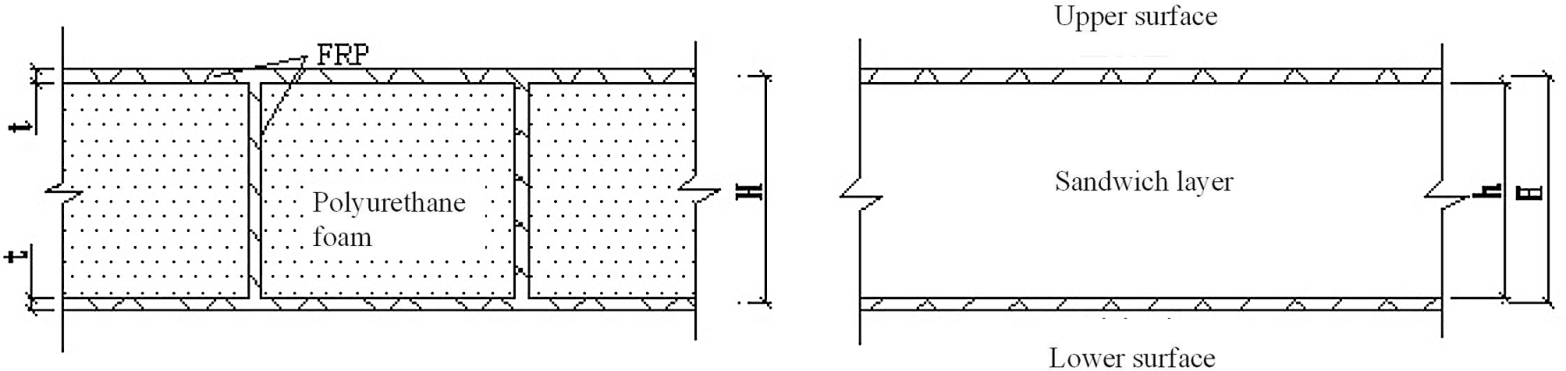
Schematic diagram of an original sandwich panel and the corresponding simulated sandwich panel.

A sandwich panel contains non-continuous core material; however, for analysis, the continuous assumption can be adopted to develop an equivalent model in which the core material is treated as continuous. Thus, the real web-reinforced composite sandwich panels are called the original sandwich panels, and the equivalent sandwich panels are called “simulated sandwich panels”. These “simulated sandwich panels” can be used in place of the original sandwich panels in mechanical analyses (Fig 12).

The continuous assumption requires the following basic hypotheses regarding the original sandwich panels.

(1) The upper face sheet and lower face sheet can sustain the in-plane force and the transverse shearing force.
(2) The polyurethane core material can sustain only the transverse shearing force and does not participate in the overall bending.
(3) Webs can sustain both shear force and bending moments. According to the classical plate theory, the in-web strain is triangularly distributed along the height of the section. Its value is zero at the neutral axis. The greater the distance from the neutral axis, the larger the strain becomes. The value reaches a maximum where the upper and lower face sheet join together, which is nearly equivalent to the strain of the upper and lower face sheet. For simplicity, suppose that the stress distribution within the web is as follows: the strain on the upper third of the web is equal to the strain on the upper face sheet, the strain on the lower third of the web is equal to the strain on the lower face sheet, and the strain on the middle third of the web is zero.

After the equivalent model has been obtained, the “simulated sandwich panel” satisfies the following basic hypotheses:

(4) The upper and lower face sheets sustain only the in-plane surface forces.
(5) The sandwich layer sustains only the transverse shearing force.
(6) After the deformation of the sandwich panel, a straight line originally perpendicular to the middle surface is still a straight line but is no longer perpendicular to the middle surface. Instead, the line has intersection angles *θ*_y_and *θ*_x_ with the middle plane in the xz and yz planes, respectively.

### Equivalent stiffness analysis of the simulated sandwich panel

#### Plane stiffness of the face sheets of the simulated sandwich panel

According to the basic hypotheses, the plane stiffness [*B*] of the simulated sandwich panel’s upper (lower) face sheet is the sum of the plane stiffness [*B*]_b_ of the original sandwich panel’s upper (lower) face sheet and the equivalent plane stiffness [*B*]_w_ of the upper (lower) one-third of the web.

According to elastic mechanical theory, the plane stiffness of the face sheets of the original sandwich panel can be determined using the following formula:

$$B_{b}=\left[\begin{array}{ccc} \frac{E_{f}t}{1-\nu_{f}^{2}} & \frac{\nu_{f}E_{f}t}{1-\nu_{f}^{2}} & 0\\ \frac{\nu_{f}E_{f}t}{1-\nu_{f}^{2}} & \frac{E_{f}t}{1-\nu_{f}^{2}} & 0\\ 0 & 0 & \frac{E_{f}t}{2(1+\nu_{f})} \end{array}\right]$$ (1)

In this formula, *E*_*f*_, *V*_*f*_ and *t* are the elastic modulus, Poisson's ratio and thickness, respectively, of the GFRP face sheet of the sandwich panel. According to the basic hypotheses, the corresponding one-third of the web can sustain only the in-plane tension, and its stress is equal to the strain on the face sheet; therefore, the plane force that it can sustain is

$$\left[\begin{array}{c} N_{x}\\ N_{y}\\ N_{xy} \end{array}\right]=\left[\begin{array}{ccc} E_{w}t_{ws} & 0 & 0\\ 0 & E_{w}t_{ws} & 0\\ 0 & 0 & 0 \end{array}\right]\left[\begin{array}{c} \varepsilon_{x}\\ \varepsilon_{y}\\ \varepsilon_{xy} \end{array}\right]=B_{w}\left[\begin{array}{c} \varepsilon_{x}\\ \varepsilon_{y}\\ \varepsilon_{xy} \end{array}\right]$$ (2)

In this formula, *N*_x_, *N*_y_, and *N*_xy_ represent the axial loads in the x and y directions and the in-plane shear load on the GFRP face sheet. *ε*_x_, *ε*_y_, and *ε*_xy_ represent the positive strains on the GFRP face sheet within the plane in the x and y directions and the in-plane shear strain, respectively. *E*_w_ is the elastic modulus of the web. *t*_ws_ is the equivalent thickness of one third of the height of the web, and [*B*_w_] is the equivalent in-plane stiffness of the web.

Therefore, the plane stiffness *[B]* of the face sheet of the simulated sandwich panel is

$$B=B_{b}+B_{w}=\left[\begin{array}{ccc} \frac{E_{f}t}{1-\nu_{f}^{2}}+E_{w}t_{ws} & \frac{\nu_{f}E_{f}t}{1-\nu_{f}^{2}} & 0\\ \frac{\nu_{f}E_{f}t}{1-\nu_{f}^{2}} & \frac{E_{f}t}{1-\nu_{f}^{2}}+E_{w}t_{ws} & 0\\ 0 & 0 & \frac{E_{f}t}{2(1+\nu_{f})} \end{array}\right]$$ (3)

#### Shear stiffness of the face sheets of the simulated sandwich panel

According to the basic hypotheses, the sandwich layer of the simulated sandwich panel is equivalent to the polyurethane core material and the web of the original sandwich panel. Therefore, the shear stiffness of the sandwich layer of the simulated sandwich panel must be equal to the sum of the shear stiffness *C*_*p*_ of the original core material and the shear stiffness *C*_*w*_ of the web, namely,

$$C=C_{p}+C_{w}$$ (4)

The shear stiffness of each element is as follows:

$$C=(a+b)hG,\;C_{p}=ahG_{p},\;C_{w}=bhG_{w}$$ (5)

In these formulae, *a* and *b* are the clear distance between two neighboring webs and the web thickness, respectively, and *h* is the web height.

From Eqs 5 and 6, the shear modulus and shear stiffness of the sandwich layer of the simulated sandwich panel can be calculated as follows:

$$G=\frac{aG_{P}+bG_{w}}{a+b}$$ (6)

$$C=Gh=\frac{aG_{P}+bG_{w}}{a+b}h$$ (7)

### Mechanical analysis of the simulated sandwich panel

**Fig 13:**
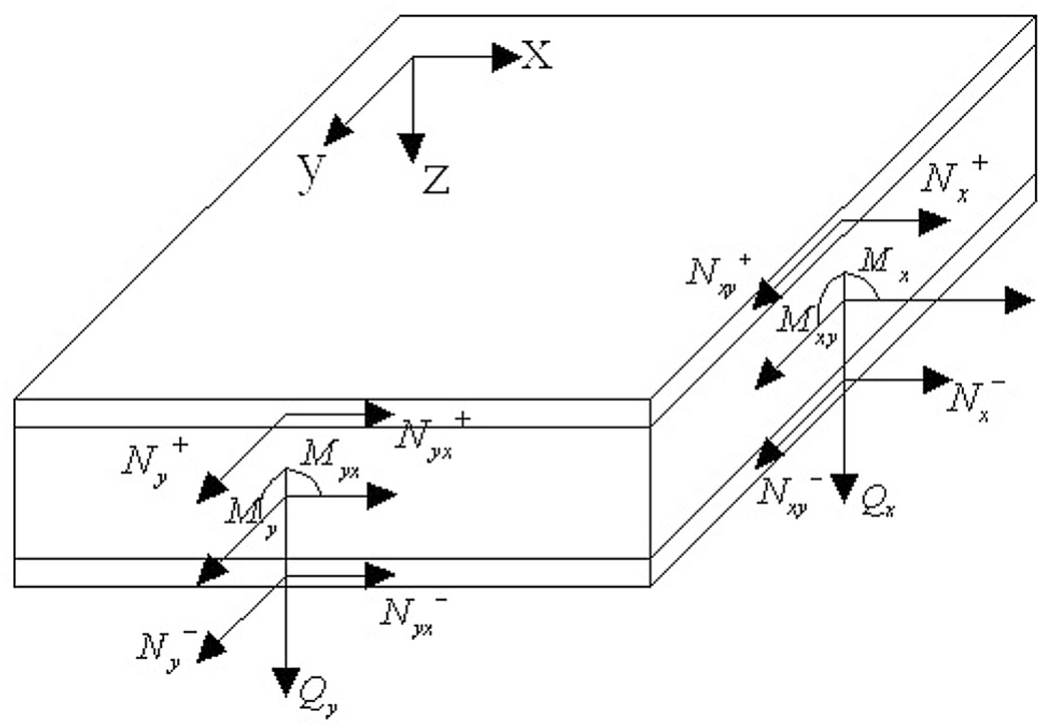
Mechanical model of the simulated sandwich panel.

The mechanical analysis model of the simulated sandwich panel is depicted in Fig 13. Based on this mechanical model, elastic mechanics theory can be adopted for the mechanical analysis of the simulated sandwich panel.

*N*_x_, *N*_y_, and *N*_xy_ are the axial loads on the GFRP face sheet in the plane in the x and y directions and the shear load, respectively. The superscript + represents the upper face sheet, and the superscript–represents the lower face sheet. *M*_x_ and *M*_xy_ represent the bending moments of the section perpendicular to the x axis along the x and y directions, respectively. *M*_y_ and *M*_yx_ represent the bending moments of the section perpendicular to the y axis along the y and x directions, respectively. *Q*_x_ and *Q*_y_ represent the shear forces on the sides perpendicular to the x and y axes, respectively, in the z direction.

#### Geometric equation

The in-plane strain analysis of the face sheet is as follows:

According to basic hypothesis 1, the displacements *u*^+^ and *v*^+^ of each point on the upper face sheet along the x and y axes are

$$u^{+}=-\frac{h+t}{2}\theta_{x}-(z-\frac{h+t}{2})\frac{\partial w}{\partial x},\;v^{+}=-\frac{h+t}{2}\theta_{y}-(z-\frac{h+t}{2})\frac{\partial w}{\partial y}$$ (8)

In these expressions, *θ*_x_ and *θ*_y_ are the intersection angles between the straight line originally perpendicular to the middle plane and the middle plane in the *yz* and *xz* planes, respectively, and *w* is the z displacement of the middle plane.

Therefore, the in-plane strain on the upper face sheet is

$$\left[\varepsilon^{+}\right]={\left[\begin{array}{ccc} \varepsilon_{x} & \varepsilon_{y} & \varepsilon_{xy} \end{array}\right]}^{T}=-\frac{h+t}{2}{\left[\begin{array}{ccc} \frac{\partial \theta_{x}}{\partial x} & \frac{\partial \theta_{y}}{\partial y} & \frac{\partial \theta_{x}}{\partial y}+\frac{\partial \theta_{y}}{\partial x} \end{array}\right]}^{T}=-\frac{h+t}{2}\left[\chi \right]$$ (9a)

Similarly, the in-plane strain on the lower face sheet is

$$\left[\varepsilon \right]=\frac{h+t}{2}\left[\chi \right]$$ (9b)

According to the basic hypotheses, the displacements *u*_cor_, *v*_cor_, and *w*_cor_ of the sandwich layer in the x, y and z directions are

$$u_{cor}=-z\theta_{x}\;,\;v_{cor}=-z\theta_{y}\;,\;w_{cor}=w$$ (10)

Therefore, the traverse shear strain on the sandwich layer is

$$\left[\gamma_{cor}\right]={\left[\gamma_{xz}\;\gamma_{xz}\right]}^{T}={\left[\frac{\partial w}{\partial x}-\theta_{x}\;\frac{\partial w}{\partial y}-\theta_{y}\right]}^{T}$$ (11)

#### Physical equation

According to the basic hypotheses, the bending moments of the simulated sandwich panel are provided by the upper and lower planes. The torque of the upper and lower face sheets with respect to the neutral axis is the bending moment *[M]*; therefore,

$$\left[M\right]=\left[N^{+}\right]\cdot \frac{h+t}{2}-\left[N^{-}\right]\cdot \frac{h+t}{2}=-\frac{{\left(h+t\right)}^{2}}{2}\left[B\right]\left[\chi \right]=\left[D\right]\left[\chi \right]$$ (12)

According to the basic hypotheses, the shear force [*Q*] on the simulated sandwich panel is sustained by the sandwich layer of the original sandwich panel; therefore, the shear force [*Q*] is

$$\left[Q\right]=\left[C\right]\left[\gamma_{cor}\right]=\frac{aG_{p}+bG_{w}}{a+b}h{\left[\frac{\partial w}{\partial x}-\theta_{x}\;\frac{\partial w}{\partial y}-\theta_{y}\right]}^{T}$$ (13)

#### Equilibrium equation

When the random lateral load that acts on the board *q* = *q* (*x*, *y*), the equilibrium equation of the simulated sandwich panel is

$$\frac{\partial M_{x}}{\partial x}+\frac{\partial M_{xy}}{\partial y}-Q_{x}=0\;,\;\frac{\partial M_{y}}{\partial y}+\frac{\partial M_{xy}}{\partial x}-Q_{y}=0\;,\;\frac{\partial Q_{x}}{\partial x}+\frac{\partial Q_{y}}{\partial y}+q=0$$ (14)

Eqs 12 and 13 can be substituted into Eq 14 to derive

$$D_{11}\frac{\partial^{2}\theta_{x}}{\partial x^{2}}+D_{33}\frac{\partial^{2}\theta_{x}}{\partial y^{2}}+(D_{12}+D_{33})\frac{\partial^{2}\theta_{y}}{\partial xy}-C(\theta_{x}-\frac{\partial w}{\partial x})=0$$ (15a)

$$D_{22}\frac{\partial^{2}\theta_{y}}{\partial y^{2}}+D_{33}\frac{\partial^{2}\theta_{y}}{\partial x^{2}}+(D_{21}+D_{33})\frac{\partial^{2}\theta_{x}}{\partial xy}-C(\theta_{y}-\frac{\partial w}{\partial uy})=0$$ (15b)

$$C(\frac{\partial^{2}w}{\partial x^{2}}+\frac{\partial^{2}w}{\partial y^{2}}-\frac{\partial \theta_{x}}{\partial x}-\frac{\partial \theta_{y}}{\partial y})+q=0$$ (15c)

A new function *Φ*is introduced such that *θ*_x_, *θ*_y_ and *w* satisfy the following relations:

$$\theta_{x}=(\frac{D_{12}+D_{33}-D_{22}}{C}\frac{\partial^{3}}{\partial x\partial^{2}y}-\frac{D_{33}}{C}\frac{\partial^{3}}{\partial^{3}x}+\frac{\partial }{\partial x})\Phi$$ (16a)

$$\theta_{y}=(\frac{D_{21}+D_{33}-D_{11}}{C}\frac{\partial^{3}}{\partial y\partial^{2}x}-\frac{D_{33}}{C}\frac{\partial^{3}}{\partial^{3}y}+\frac{\partial }{\partial y})\Phi$$ (16b)

$$\begin{array}{l} w=(\frac{D_{11}D_{22}-D_{12}^{2}-2D_{12}D_{33}}{C^{2}}\frac{\partial^{4}}{\partial^{2}x\partial^{2}y}+\frac{D_{11}D_{33}}{C^{2}}\frac{\partial^{4}}{\partial^{4}x}+\frac{D_{22}D_{33}}{C^{2}}\frac{\partial^{4}}{\partial^{4}y}\\ \;-\frac{D_{11}+D_{33}}{C}\frac{\partial^{2}}{\partial^{2}x}-\frac{D_{22}+D_{33}}{C}\frac{\partial^{2}}{\partial^{2}y}+1)\Phi \end{array}$$ (16c)

When Eq 16 is substituted into Eq 15, Eqs 15a and 15b are automatically tenable, whereas Eq 15c can be simplified to

$$\begin{array}{l} (-\frac{D_{11}D_{33}}{C}\frac{\partial^{6}}{\partial^{6}x}-\frac{D_{22}D_{33}}{C}\frac{\partial^{6}}{\partial^{6}y}-\frac{D_{11}D_{22}+D_{11}D_{33}-D_{12}^{2}-2D_{12}D_{33}}{C}\frac{\partial^{6}}{\partial^{4}x\partial^{2}y}\\ \;-\frac{D_{11}D_{22}+D_{12}D_{33}-D_{12}^{2}-2D_{12}D_{33}}{C}\frac{\partial^{6}}{\partial^{2}x\partial^{4}y}+D_{11}\frac{\partial^{4}}{\partial^{4}x}+D_{22}\frac{\partial^{4}}{\partial^{4}y}\\ \;+2(2D_{33}+D_{12})\frac{\partial^{4}}{\partial^{2}x\partial^{2}y})\Phi =q \end{array}$$ (16)

Therefore, a six-order partial differential equation represented by the new displacement function *Φ*, in reference to the original sandwich panel, can be used to model the simulated sandwich panel.

### Solution for panels with four simply supported edges

The boundary conditions for a rectangular sandwich panel with simple support for all four edges are as follows:

$$x=0\;\text{and}\;x=L,\;w=0,\;\theta_{y}=0,\;\frac{\partial \theta_{x}}{\partial x}=0$$ (17a)

$$y=0\;\text{and}\;y=L,\;w=0,\;\theta_{x}=0,\;\frac{\partial \theta_{y}}{\partial y}=0$$ (17b)

The lateral load *q = q*(*x*, *y*), which is randomly distributed over the board, can be expanded by means of the double trigonometric function as follows:

$$q(x,y)=\sum_{m=1}^{\infty }\sum_{n=1}^{\infty }Q_{mn}\text{cos}\frac{m\pi x}{L}\text{cos}\frac{n\pi y}{L}$$ (18)

If the load that is distributed over the board is uniformly distributed, that is, when *q* (*x*, *y*) = *q*_0_,

$$Q_{mn}=\frac{4}{\pi^{2}mn}q_{0}(1-\;\text{cos}\;m\pi )(1-\;\text{cos}\;n\pi )$$

When the concentrated load *P* acts on the center of the board,

$$Q_{mn}=\frac{4}{L^{2}}P\;\text{sin}\frac{m\pi }{2}\text{sin}\frac{n\pi }{2}$$

In addition, the displacement function *Φ* is expanded by means of the double trigonometric function as follows:

$$\Phi =\sum_{m=1}^{\infty }\sum_{n=1}^{\infty }Q_{mn}A_{mn}\;\text{cos}\frac{m\pi x}{L}\text{cos}\frac{n\pi y}{L}$$ (19)

Obviously, the function *Φ* satisfies the above boundary conditions. When Eqs 18 and 19 are substituted into Eq 16, the coefficient *A*_mn_ can be determined as follows:

$$\begin{array}{l} \frac{1}{A_{mn}}=\frac{\pi^{2}}{L}(\frac{D_{11}D_{33}}{C}m^{6}+\frac{D_{22}D_{33}}{C}n^{6}+\frac{D_{11}D_{22}+D_{11}D_{33}-D_{12}^{2}-2D_{12}D_{33}}{C}m^{4}n^{2}\\ \;\frac{D_{11}D_{22}+D_{12}D_{33}-D_{12}^{2}-2D_{12}D_{33}}{C}m^{2}n^{4}+\alpha (D_{11}m^{4}+D_{22}n^{4}+2D_{12}m^{2}n^{2}+4D_{33}m^{2}n^{2})) \end{array}$$ (20)

In this expression, *α* = *L*^2^/*π*^2^. The expression for *Φ* can be substituted into the deflection expression, namely, the intersection angle expression, to derive the following:

$$\theta_{x}=\sum_{m=1}^{\infty }\sum_{n=1}^{\infty }\Delta_{1}Q_{mn}A_{mn}\;\text{sin}\frac{m\pi x}{L}\text{cos}\frac{n\pi y}{L}$$ (21a)

$$\theta_{y}=\sum_{m=1}^{\infty }\sum_{n=1}^{\infty }\Delta_{2}Q_{mn}A_{mn}\;\text{cos}\frac{m\pi x}{L}\text{sin}\frac{n\pi y}{L}$$ (21b)

$$w=\sum_{m=1}^{\infty }\sum_{n=1}^{\infty }\Delta_{3}Q_{mn}A_{mn}\;\text{cos}\frac{m\pi x}{L}\text{cos}\frac{n\pi y}{L}$$ (21c)

In these expressions, *Δ*_1_ and *Δ*_2_ are the intersection angle coefficients, and *Δ*_3_ is the deflection coefficient.

$$\Delta_{1}=\frac{L^{3}}{\pi^{3}}(\frac{D_{12}+D_{33}-D_{22}}{C}mn^{2}\frac{\pi^{2}}{L}-\frac{D_{33}}{C}m^{3}\frac{\pi^{2}}{L}-mL)$$

$$\Delta_{2}=\frac{L^{3}}{\pi^{3}}(\frac{D_{21}+D_{33}-D_{11}}{C}m^{2}n\frac{\pi^{2}}{L}-\frac{D_{33}}{C}n^{3}\frac{\pi^{2}}{L}-nL)$$

$$\begin{array}{l} \Delta_{3}=\frac{L^{3}}{\pi^{3}}(\frac{D_{11}D_{22}-D_{12}^{2}-2D_{12}D_{33}}{C^{2}}m^{2}n^{2}\frac{\pi^{3}}{L^{2}}+\frac{D_{11}D_{33}}{C^{2}}m^{4}\frac{\pi^{3}}{L^{2}}+\frac{D_{22}D_{33}}{C^{2}}n^{4}\frac{\pi^{3}}{L^{2}}\\ \;+\frac{D_{11}+D_{33}}{C}m^{2}\pi +\frac{D_{22}+D_{33}}{C}n^{2}\pi +\frac{L^{2}}{\pi }) \end{array}$$

After solving for the three displacements *θ*_x_, *θ*_y_ and *w*, they can be substituted into Eqs 12 and 13. The internal forces *[M]* and [*Q*] can then be calculated. In the expression of the deflection coefficient *Δ*_3,_ only the last term, which represents the deflection deformation that is caused by the bending effect, is irrelevant to the shear stiffness. However, all the other terms, which represent the deflection deformation that is caused by the shear effect, are relevant to the shear stiffness *C*.

### Comparison with experimental results

**Fig 14:**
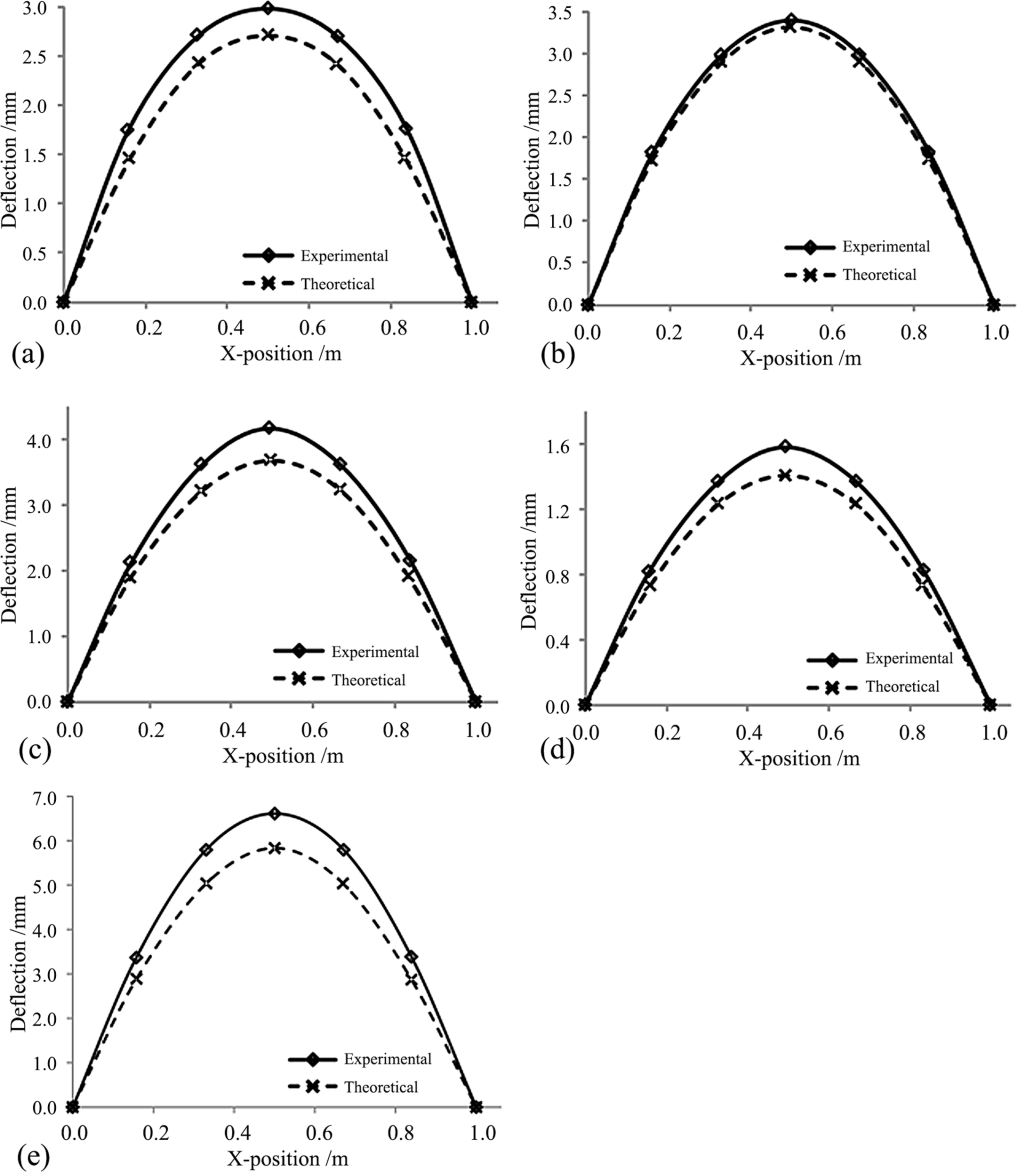
Calculation of specimen deflections and comparison with the experimental values. (a) SX75-75 deflection. (b) SX75-125 deflection. (c) SX75-175 deflection. (d) SX100-75 deflection. (e) SX50-75 deflection.

Fig 14 shows the contrast between the experimental and theoretical values of the vertical deflection at the central point of the sandwich panel’s underside under a uniformly distributed load of 10.08 kN/m^2^. The results show that the theoretical values for all the experimental specimens were slightly smaller than the experimental values, which indicates that the mechanical model herein overestimated the stiffness of the actual specimens. However, the errors between the theoretical values and experimental values were all small, and the relative error (experimental-theoretical value/theoretical value) of the deflection at the central point of the board was 10%, which indicates that the calculations that were proposed herein can adequately calculate the deflection of the sandwich panel. In addition, Table 5 shows the deflections that were caused by the bending effect and shear effect. When the total heights of the web were kept constant (at 75 mm) and the web spacing was reduced from 175 mm to 75 mm, the total deflection decreased from 4.14 mm to 2.98 mm, down by 28%; additionally, the deflection that was caused by the bending effect and the shear effect decreased from 1.65 mm and 2.03 mm to 1.44 mm and 1.27 mm, down by 13.1% and 37.2%, respectively. The results show that the deflection that was caused by the shear effect when reducing the web spacing decreased much more than that from the bending effect. Additionally, when the web spacing was kept constant and the web height increased from 50 mm to 100 mm, the ratio of the deflection deformation that was caused by the bending effect to the total deflection decreased from 69.9% to 39.9%. The results show that increasing the web thickness can effectively decrease the deflection. However, the ratio of the deflection that was caused by the shear effect to the total deflection increased significantly, even exceeding the deformation from the bending effect.

**Table 5:** Contrast in vertical deflections at the central point of the sandwich panels (uniformly distributed load q = 10.08kN/^2^).

Serial No. of specimen	Experimental value	Theoretical value			Relative error* (%)
	Central point deflection *f*_0_ (mm)	Bending-caused deflection *f*_M_ (mm)	Shear-caused deflection *f*_S_ (mm)	Central point deflection *f*_0*T*_ (mm)	
SX75-75	2.98	1.44 (53.0%)**	1.27 (47.0%)	2.71	9.1
SX75-125	3.39	1.62 (49.1%)	1.68 (50.9%)	3.30	2.7
SX75-175	4.14	1.65 (44.9%)	2.03 (55.1%)	3.68	11.1
SX50-75	6.61	4.08 (69.9%)**	1.75 (30.1%)	5.83	11.8
SX100-75	1.58	0.56 (39.9%)	0.85 (60.1%)	1.41	10.8

*: Relative error = (*f*_0_—*f*_0T_) / *f*_0_×100%

** The percentage within the bracket indicates the ratio of the deflection from the bending effect or the shear effect to the total deflection.

The calculated values of the maximum tensile stress of the lower face sheet that correspond to the equivalent yield point under a concentrated load are indicated in Table 4. When the web spacing values were the same (75 mm), the maximum tensile stress of the lower face sheet of the ordinary sandwich panel SF75 was only 22.8 MPa, approximately 7.1% of its ultimate tensile strength (322.9 MPa). When the web-reinforced sandwich panel reached the equivalent yield load, the maximum tensile stress of the lower face sheet increased significantly with a reduction in the web spacing. When the web spacing reduced from 175 mm to 75 mm, the maximum tensile stress increased from 40.2 MPa to 157.9 MPa, and its ratio to the ultimate tensile strength of the lower face sheet increased from 12.4% to 48.9%, which indicates that a reduction in the web spacing can effectively increase the service efficiency of the panel material.

## Conclusions

The vacuum infusion process was used to fabricate 5 web-reinforced composite sandwich panels and 1 ordinary composite sandwich panel, and each panel was simply supported on all four edges. Loading tests were performed with uniformly distributed loads and central single-point loads. Finally, the simulated sandwich panel method was used to deduce the formula for calculating the deflection deformation of a sandwich panel under a uniformly distributed load. The main conclusions of the study are as follows:

1. Adding webs to an ordinary sandwich panel can modify the mechanical behavior and failure modes of the sandwich panel under concentrated loads. Ordinary web sandwich panels without web reinforcement exhibit no yield stage during the loading process, and brittle failure occurs immediately at the maximum load. The observed failure mode is interfacial debonding failure. By contrast, a reinforced composite sandwich panel first exhibits vertical and horizontal cracks during the loading process, and such a sandwich panel exhibits overall plastic behavior. When the ultimate load is reached, the top face sheet is damaged. Upon damage, reinforced sandwich panels exhibit good ductility, and the maximum ductility coefficient is 2.73 times that of webless sandwich panels.
2. The web spacing has a significant effect on the elastic deflection of reinforced composite sandwich panels. Under a uniformly distributed load, for specimens with a web height of 75 mm and web spacing values of 175 mm, 125 mm, and 75 mm, the midspan deflections were 64.2%, 52.6% and 46.2% that of the webless component, respectively. These results show that placing webs in webless components and reducing the spacing between webs in web-reinforced components can effectively increase the flexural rigidity of these components and reduce their vertical deformation.
3. Web spacing and web height have a significant influence on the bearing capacity of a sandwich panel under a concentrated load. The equivalent yield load and limit load of the specimen with a web spacing of 75 mm were found to be 3.88 and 4.63 times those of the sandwich panel without web reinforcement, respectively, and the equivalent yield load and limit load of the specimen with a web height of 100 mm were twice those of the specimen with a web height of 50 mm.
4. The simulated sandwich panel method was used to perform a deformation analysis of a web-reinforced composite sandwich panel with four simply supported edges, and the deflection calculation formulae were deduced for uniformly distributed loading conditions. The uniformly distributed load function and displacement function were solved by expansion into double trigonometric series, and the maximum error between the calculated results and the experimental results was less than 15%.

In this study, the bending mechanical behavior of web-reinforced composite sandwich panels under two-way bending was examined for the first time, and the influence of the web spacing and height on the mechanical properties of such a panel was determined. The formulae for calculating the deflection deformation based on the simulated sandwich panel method yield results that are in good agreement with the test results. This study provides a new approach to improving the mechanical properties of sandwich panels and can serve as a reference for the application of sandwich panels of this type in structures such as floor slabs and roofs.

This study also has certain limitations. Each specimen was a square panel, with equal dimensions in the horizontal and vertical directions, which is a special case of two-way slabs. In practical engineering applications, most floor slabs are rectangular panels (with a length-width ratio >1), and the stress states in the length and width directions are not the same. Further studies on the horizontal and vertical arrangements of webs will be required to optimize their effect on the stress states of sandwich panels.
